# Korean Red Ginseng Suppresses the Expression of Oxidative Stress Response and NLRP3 Inflammasome Genes in Aged C57BL/6 Mouse Ovaries

**DOI:** 10.3390/foods9040526

**Published:** 2020-04-22

**Authors:** Sungwoo Chei, Hyun-Ji Oh, Hoon Jang, Kippeum Lee, Heegu Jin, Youngsok Choi, Boo-Yong Lee

**Affiliations:** 1 Department of Food Science and Biotechnology, College of Life Science, CHA University, Seongnam, Kyonggi 13488, Korea; sungwoochei@gmail.com (S.C.); guswl264@naver.com (H.-J.O.); joy4917@hanmail.net (K.L.); heegu94@hanmail.net (H.J.); 2 Department of Stem Cell and Regenerative Biotechnology, Humanized Pig Research Center (SRC), Konkuk University, Seoul 05029, Korea; sojiro38@naver.com

**Keywords:** Korean ginseng extract, oxidative stress response, NLRP3 inflammasome, aging, ovary, subfertility

## Abstract

Female infertility and subfertility have been increasing in prevalence worldwide. One contributing factor is ovarian function, which is highly age-dependent. Korean red ginseng is widely used as an herbal medicine and has many beneficial properties. We aimed to determine the effect of the Korean red ginseng saponin fraction (KRGSF) on ovarian function in female C57BL/6 mice. Ovaries were isolated from 6- and 12-month-old female mice and treated with KRGSF, and then RNA was extracted and microarray analysis was performed. The expression of key genes was subsequently verified using quantitative RT-PCR. Aging markedly increased the expression of genes encoding oxidative stress factors and NLRP3 inflammasome components, but the expression of these genes was significantly reduced by KRGSF treatment. Thus, the reduction in ovarian health with age is associated with greater oxidative stress response and inflammation, but KRGSF treatment may limit these age-related changes.

## 1. Introduction

Average life expectancy has increased worldwide alongside economic growth and the progression of medical technology, and this increase in life expectancy, along with cultural and social factors, has led people to marry and give birth later than previously [1,2,3,4]. This trend has emphasized the effect of aging on the fertility of otherwise healthy couples. Aging is associated with a reduction in metabolic activity and the accumulation of waste products in tissues and cells, which causes errors in molecular processes and can result in diseases such as cancer, dementia, heart disease, and subfertility [5,6,7,8]. Reproductive capacity diminishes with age, and, in particular, maternal age and ovarian health are crucial determinants of fertility [9,10].

Oxidative stress involves a rapid increase in harmful reactive oxygen species (ROS) in cytoplasm, which have adverse cellular effects. It is considered to be an imbalance between the production of ROS and their removal by protective mechanisms, which can cause inflammation. Isocitrate dehydrogenase (*Idh1*) catalyzes the decarboxylation of isocitrate to form α-ketoglutarate and CO_2_, and generates NADPH from NADH^+^, which provides an antioxidant effect to counter the production of ROS [11]. Consistent with this, we previously showed that *Idh* mutant *Drosophila* has lower cellular NADPH concentration, greater ROS production, and hypersensitivity to oxidative stress.

The activation of inflammasomes is an important signaling mechanism in the innate immune system. Inflammasomes are classified according to their sensor protein, and one of the best studied, the NOD-like receptor protein 3 (NLRP3) inflammasome, is a multi-protein scaffolding complex [12,13]. Activation of the NLRP3 inflammasome requires the adaptor protein apoptosis-associated speck-like protein containing a C-terminal cascade recruitment domain (ASC) and leads to the cleavage, and thereby activation, of pro-caspase-1. This caspase-1 activation is important for the maturation and secretion of the cytokines interleukin (IL)-1β and IL-18.

It is very important to protect the ovaries from aging-related oxidative stress and inflammasome activation to increase the chance of a successful pregnancy [6]. However, the precise mechanisms underlying age-related female subfertility have remained unclear, despite a great deal of research having been conducted in this area, and no therapeutic agents have been developed. To improve understanding of aging-related female subfertility, it is necessary to analyze how gene expression in the ovary changes with age, and to elucidate the associated regulatory mechanisms.

Previous studies have shown that ginseng is a beneficial natural product and can be used to treat various metabolic diseases [14,15,16]. Ginseng contains many saponins, including Rb1, Rg3, and Rc, but the saponin content varies according to the extraction method used [17,18,19]. Many studies have demonstrated that ginseng-derived saponins can ameliorate metabolic diseases, such as obesity, diabetes, and dyslipidemia [20]. Ginseng extract has also been shown to improve male reproductive capacity by increasing sperm motility and number [19,20,21,22,23]. However, the effects of ginseng extract on female fertility have not been characterized. Therefore, in the present study, we aimed to determine the effects of ginseng-derived saponins on age-related female subfertility by profiling gene expression in the ovaries of young and older mice, some of which were treated with Korean red ginseng saponin fraction (KRGSF).

## 2. Materials and Methods 

### 2.1. Preparation of Ginseng Saponin Fraction

Korean red ginseng saponin fraction (KRGSF) was prepared from 6-year-old Korean red ginseng water extract (Korea Ginseng Corp., Daejeon, Korea) as described previously [24,25]. In brief, the Korean red ginseng was extracted with water at about 95 °C for 2 h, and this process was repeated three times. The resultant water extracts were filtered with filter paper. Saponin fraction of water extract was collected with butanol extraction. Saponin fraction was concentrated by vacuum evaporation and freeze-dried. The saponin fraction powder was obtained. 

Ginsenoside composition was analyzed by HPLC as described previously [13]. The total ginsenoside composition of each sample was examined three times. The 99% purity of ginsenoside standards were used in this study. A Waters 1525 binary HPLC system (Waters, MA, USA), fitted with a Eurospher 100-5 C18 column (Knauer, 3 × 250 mm, Germany) was used. The mobile phase was the mixture of acetonitrile and distilled water. The content of acetonitrile was progressively increased from 17% to 25% (25 min), 25% to 40% (50 min), 40% to 60% (105 min), 60% to 100% (110 min), and finally adjusted from 100% to 17% (125 min, lasting for 10 min) again. The operating temperature was set to room temperature, and the flow rate was 0.8 mL/min. The elution profile on a chromatogram was gained by using a UV/VIS detector at 203 nm (Waters 2487 dual λ absorbance detector, Boston, MA, USA). 

### 2.2. Chemicals and Reagents

Alpha-MEM, penicillin-streptomycin (P/S, 100×), charcoal stripped fetal bovine serum (CS-FBS), and TRIzol reagent were purchased from Thermo Fisher Scientific (Waltham, MA, USA). ITS-Plus Media Supplement was purchased from R&D Systems (Minneapolis, MN, USA). Unless noted otherwise, all chemicals were purchased from Sigma Chemical Co. (St. Louis, MO, USA).

### 2.3. Animal Experiments

Six-month-old and twelve-month-old female C57BL/6 mice were purchased from Orient Bio Company (Seongnam, Korea) and housed at 20 ± 3 °C under a 12 h light–dark cycle. The Institutional Animal Care and Use Committee of CHA University (IACUC No. 150047) approved all animal experiments, and all experiments were performed according to the guidelines for the care and use of laboratory animals. All mice were sacrificed by CO_2_ inhalation. Ten pairs of ovaries from 6-month-old and 12-month-old mice were isolated, and each set was randomly divided into two groups (6-month old ovaries data not shown).

### 2.4. Culture and KRGSF Treatment of Mouse Ovaries 

Mouse ovaries were prepared and cultured in vitro using previously reported methods [26]. The ovaries were isolated from experimental mice and gently washed with PBS. Tissues were incubated in Dulbecco’s modified Eagle’s medium (DMEM) containing 10% fetal bovine serum (FBS) and 1% penicillin/streptomycin (P/S) with or without KRGSF (10mg/mL) for 24 h at 37 °C and 5% CO_2_ in a humidified incubator.

### 2.5. Total RNA Isolation and Microarray Analysis

Ovaries were obtained from C57BL/6 mice and total RNA was isolated using TRIzol reagent in accordance with the manufacturer’s protocol. Microarray analysis was performed using Mouse Gene ST arrays (Affymetrix, Seoul, Korea). Expression values were computed from the raw data, and gene set enrichment analysis was applied to interpret expression profiles (Biocore, Seoul, Korea). The gene ontology (GO) performed enrichment analysis on gene sets (GSE 144954).

### 2.6. qRT-PCR

Ovaries were homogenized in 500 μL of TRIzol reagent, and total RNA was extracted according to the manufacturer’s protocol. cDNA was prepared from 1 μg of total RNA using a Bio-Rad RT-PCR system (CA, USA). Gene expression levels were quantified using Power SYBR Green (Mannheim, Germany) and a Bio-Rad CFX96 Real-Time Detection System (CA, USA). The forward and reverse primers used for qRT-PCR are listed in the Table 1. The related mRNA gene expression was obtained by normalization against the control gene (18S) and calculated by the comparative 2^−∆∆CT^ method.

### 2.7. Statistical Analysis

Differences between multiple groups were evaluated using one-way analysis of variance (ANOVA) or Student’s *t*-test, followed by Duncan’s multiple range test, using SPSS software (IBM, Chicago, IL, USA). Significantly different values (*p* < 0.05) are indicated with different letters.

## 3. Results

### 3.1. Preparation and Composition of KRGSF

**Figure 1:**
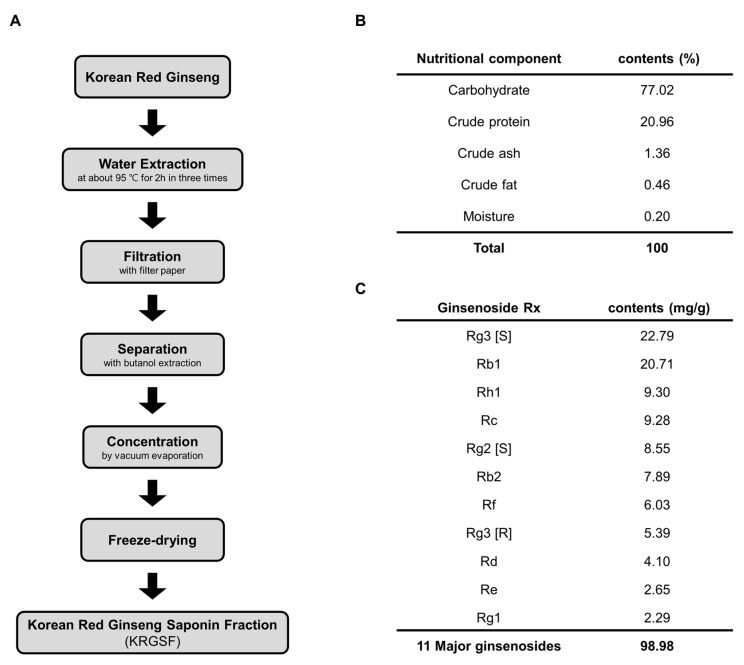
The nutritional composition and ginsenoside content of the Korean red ginseng saponin fraction. (**A**) Scheme for the preparation of the KRGSF. (**B**) The nutrient composition of Korean red ginseng saponin fraction (KRGSF). (**C**) The ginsenoside content of the KRGSF.

A saponin fraction was prepared from 6-year-old Korean red ginseng. As shown in Figure 1A, the Korean red ginseng was extracted with water at about 95 °C for 2 h, and this process was repeated three times. The resultant water extracts were filtered with filter paper. The saponin fraction of the water extract was collected with butanol extraction. The saponin fraction was concentrated by vacuum evaporation and freeze-dried. The nutritional components of the saponin fraction powder were then characterized (Figure 1B). The nutrient composition of KRGSF was as follows: carbohydrate (77.02%), crude protein (20.96%), crude ash (1.36%), crude fat (0.46%), and water (0.20%). In addition, the major chemical components were identified using high-performance liquid chromatographic analysis (Figure 1C). The ginsenoside content of the ginseng saponin fraction was as follows: RG3 [S] (22.79 mg), Rb1 (20.71 mg), Rh1 (9.30 mg), Rc (9.28 mg), RG2 [S] (8.55 mg), Rb2 (7.89 mg), Rf (6.03 mg), Rg3 [R] (5.39 mg), Rd (4.10 mg), Re (2.65 mg), and Rg1 (2.29 mg).

### 3.2. Age and KRGSF Treatment Affect the Ovarian mRNA Expression Profile

**Figure 2:**
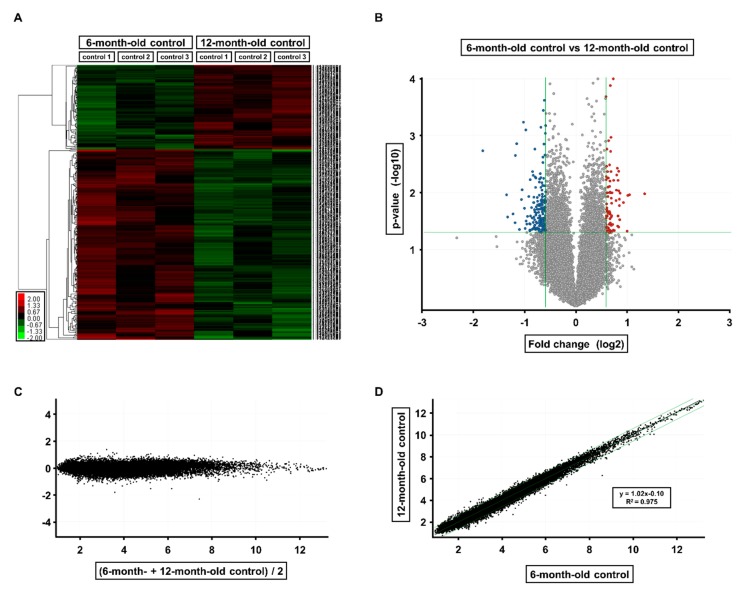
Gene expression in ovaries from 6-month-old and 12-month-old C57BL/6 mice: (**A**) hierarchical clustering and heat map analysis. A heat map of significantly differentially expressed (>1.5-fold) genes is shown. Red dots represent higher expression and green dots represent lower expression at 12 months of age. Volcano plot (**B**) and scatter plots (**C**,**D**) of the microarray analysis data.

**Figure 3:**
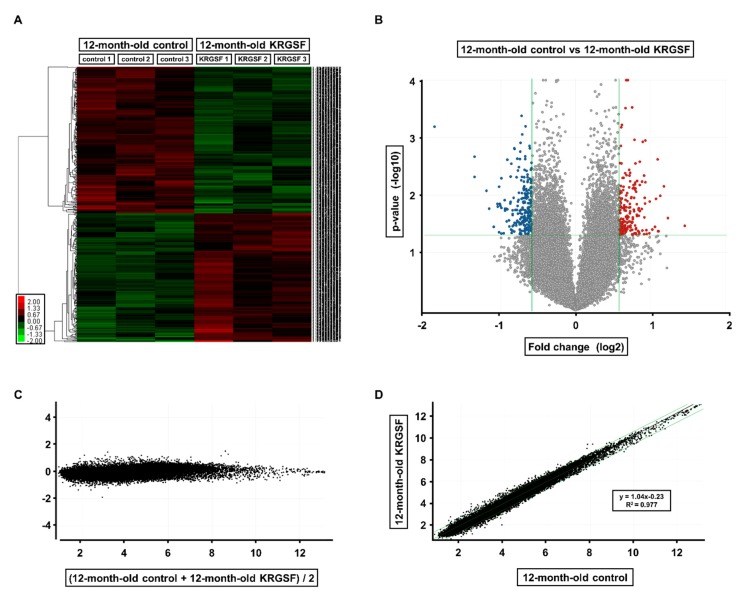
Gene expression in the ovaries of KRGSF-treated and untreated 12-month-old C57BL/6 mice: (**A**) hierarchical clustering and heat map analysis. A heat map of significantly differentially expressed (1.5-fold) genes is shown. Red dots represent higher expression and green dots represent lower expression at 12 months of age. Volcano plot (**B**) and scatter plots (**C**,**D**) of the microarray analysis data.

The effects of age and KRGSF on gene expression in the ovary were analyzed using microarrays (Figure 2 and Figure 3). We compared 6-month-old untreated, 12-month-old untreated, and 12-month-old KRGSF-treated mice. Each treatment group consisted of three samples that were selected on the basis of high RNA purity.

To determine the effect of aging on the ovaries of C57BL/6 mice, we compared gene expression in the 12-month-old untreated group with that in the 6-month-old untreated group (Figure 2). The expression of 236 genes was >1.5-fold higher and the expression of 580 genes was >1.5-fold lower in 12-month-old mice vs. 6-month-old mice. Of these, the expression of 81 genes was significantly higher and the expression of 181 genes was significantly lower (Figure 2A). The volcano plot of the microarray data shows genes with >1.5-fold higher expression as red dots, genes with >1.5-fold lower expression as blue dots, and genes with similar expression as gray dots (Figure 2B). In the M vs. A plot, a positive value on the y-axis indicates high expression relative to that of 6-month-old mice, a negative value indicates low expression relative to that of 6-month-old mice, and a value close to 1 indicates similar expression (Figure 2C). In the intensity scatter plot, the gray line indicates a 1.5-fold difference in expression; points above demonstrate the distribution of genes with higher expression in 12-month-old mice, and points below demonstrate the distribution of genes with lower expression in 12-month-old mice (Figure 2D).

We then determined the effect of KRGSF treatment on the ovaries of aged C57BL/6 mice (Figure 3). We found that 369 genes had >1.5-fold higher expression, and 580 genes had >1.5-fold lower expression in treated mice. Of these, the expression of 194 genes was significantly higher and that of 222 genes was significantly lower (Figure 3A). After gene expression was normalized, a volcano plot, an M vs. A plot, and an intensity scatter plot were generated to visualize the relative fold differences in gene expression resulting from KRGSF treatment (Figure 3B–D). In summary, the ovarian gene expression pattern of mice was affected by both aging and KRGSF treatment.

### 3.3. Gene Ontology Enrichment Analysis to Identify the Functions of Genes Affected by KRGSF Treatment

**Figure 4:**
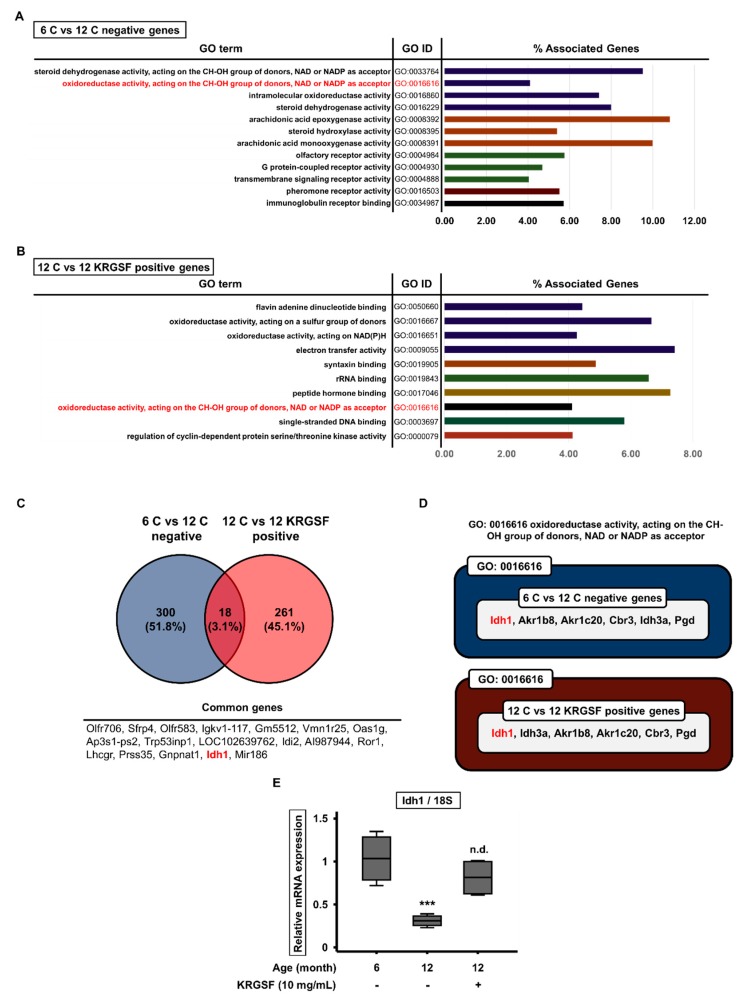
Molecular functional classification of the differentially expressed genes’ gene ontology (GO) term enrichment analysis was performed using the Clue Go plugin for Cytoscape software. (**A**) The GO molecular functions of the differentially expressed genes in ovaries from 12-month-old and 6-month-old untreated mice. (**B**) The GO molecular functions of the differentially expressed genes in ovaries from 12-month-old KRGSF-treated and untreated mice. GO terms shown in red were affected by both aging and KRGSF treatment. (**C**) Comparison of the age-related and KRGSF-related differential gene expression. (**D**) The GO term GO:0016616 was affected by both aging and KRGSF. Genes shown in red were affected by both. (**E**) Quantitative RT-PCR analysis of ovarian *Idh1* expression. 18S was used as a reference gene. Mean ± SEM, *n* = 4, *** *p* < 0.005 compared with the 6-month-old untreated group. n.d. (no difference).

The microarray analysis identified numerous genes that were significantly differentially expressed among 6-month-old untreated, 12-month-old untreated, and 12-month-old KRGSF-treated mice. We next conducted a gene ontology (GO) enrichment analysis to determine the “molecular function” terms for these genes. The Clue Go plugin for Cytoscape software was used to identify the functional groups in networks of the up- and downregulated genes. The top 12 molecular functions of the downregulated genes in 12-month-old vs. 6-month-old untreated mice included oxidoreductase activity (GO:0016616), and this was also included in the top ten molecular functions of the upregulated genes in 12-month-old KRGSF-treated vs. untreated mice (Figure 4A,B). We also found that the expression of 300 genes was lower only in 12-month-old vs. 6-month-old untreated mice, and that the expression of 261 genes was higher only in 12-month-old KRGSF-treated vs. untreated mice. There were 18 genes that were affected by both aging and KRGSF treatment, which accounted for 3.1% of the total (Figure 4C). Therefore, we focused on the molecular function of oxidoreductase activity (GO:0016616), which was affected by both aging and KRGSF treatment (Figure 4A,B), and found that *Idh1* expression was affected by both (Figure 4D). We performed quantitative RT-PCR to verify the effects of aging and KRGSF treatment on the mRNA expression of *Idh1* in the ovaries of experimental mice, and found that, compared with 6-month-old mouse ovaries, the mRNA expression of *Idh1* was significantly lower in 12-month-old mouse ovaries, but was increased by KRGSF treatment, such that it did not show a statistically significant difference from that in the 6-month-old untreated mouse ovaries (Figure 4E). These data imply that KRGSF increases the expression of genes encoding oxidoreductases, which is reduced by aging, and these genes include *Idh1*.

### 3.4. KRGSF Inhibits Age-Related Intracellular Oxidative Stress Response

**Figure 5:**
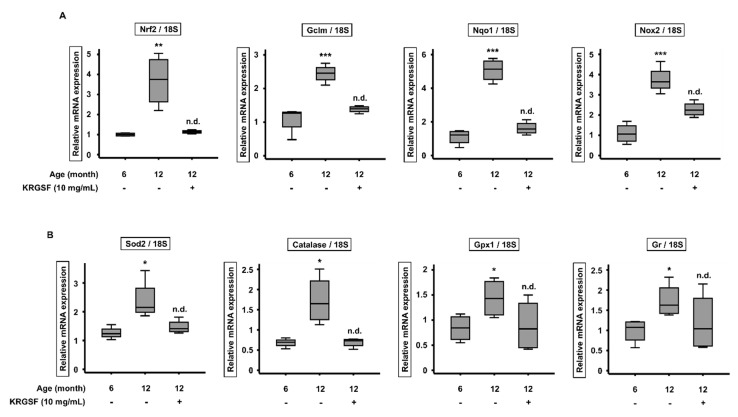
KRGSF inhibits the age-related increase in expression of intracellular oxidative stress response genes. RNA was isolated from ovaries and quantitative RT-PCR analysis was performed. (**A**) The relative mRNA expression of *Nqo1*, *Gclm*, *Nrf2*, and *Nox2* (mean ± SEM; *n* = 4). (**B**) The relative mRNA expression of *Sod2*, *Catalase*, *Gpx1*, and *Gr* (mean ± SEM; *n* = 4). 18S was used as a reference gene. * *p* < 0.05, ** *p* < 0.01, and *** *p* < 0.005 vs. the 6-month-old untreated group. n.d. (no difference).

*Idh1* is well known to inhibit intracellular oxidative stress [11]. We performed quantitative PCR to measure the expression of genes associated with oxidative stress response. Nrf2 is an antioxidative transcription factor for antioxidant genes, such as *Nqo1* and *Gclm* [11]. Compared with 6-month-old untreated mice, the mRNA expression of the genes *Nqo1*, *Gclm*, and *Nrf2*, which encode antioxidant proteins, was considerably higher in the ovaries of 12-month-old untreated mice. However, the expression of these genes was reduced by KRGSF treatment, such that there were no significant differences from the expression levels in 6-month-old untreated mice. The expression of the oxidative stress-associated gene *Nox2* showed the same tendency (Figure 5A). We also measured the expression of genes encoding antioxidant enzymes, which reduce the ROS concentration during oxidative stress. The mRNA expression of *Sod2*, *Catalase*, *Gpx1*, and *Gr* was much higher in the ovaries of 12-month-old untreated mice than in those of 6-month-old untreated mice, but the expression of all of these genes was reduced by KRGSF treatment (Figure 5B). These results suggest that KRGSF inhibits the age-related excessive accumulation of ROS and prevents oxidative stress response, which should ameliorate ovarian loss of function.

### 3.5. KRGSF Ameliorates the Age-Related Upregulation of NLRP3 Inflammasome Gene Expression

**Figure 6:**
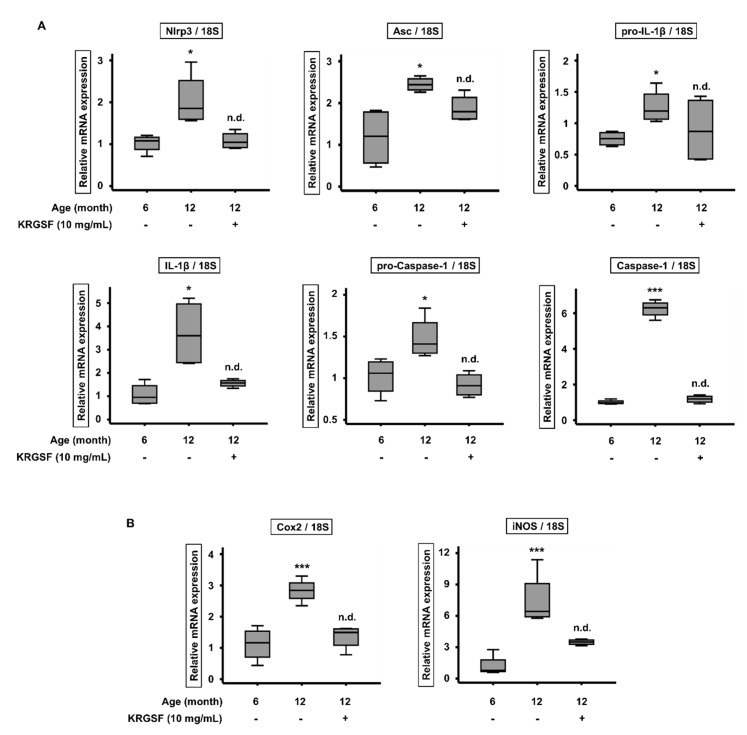
KRGSF ameliorates the aging-related increase in expression of genes encoding NLRP3 inflammasome components. RNA was isolated from ovaries and quantitative RT-PCR analysis was performed. (**A**) The relative mRNA expression of *Nlrp3*, *Asc*, pro-IL-1β, IL-1β, pro-caspase-1, and caspase-1 (mean ± SEM; *n* = 4). (**B**) The relative mRNA expression of *Cox2* and iNOS2 (mean ± SEM; *n* = 4). 18S was used as a reference gene. * *p* < 0.05 and *** *p* < 0.005 vs. the 6-month-old untreated group. n.d. (no difference).

ROS stimulates the activation of NLRP3 inflammasome [27], which is a protein complex that initiates an inflammatory response [28,29] that damages ovaries when excessive [30]. Therefore, we next determined whether KRGSF affects the expression of genes encoding NLRP3 inflammasome components by quantitative RT-PCR (Figure 6A). The mRNA expression of Nlrp3 and Asc was higher in 12-month-old than in 6-month-old untreated mice, but the expression of these genes in 12-month-old KRGSF-treated mice was not statistically significantly different from that in 6-month-old untreated mice. As expected, the mRNA expression of pro-IL-1β and IL-1β was higher in 12-month-old than in 6-month-old untreated mice. In addition, the expression of the genes encoding pro-caspase-1 and caspase-1 was higher in 12-month-old untreated mice. However, the expression of all of these genes was reduced by KRGSF treatment. We also determined the effects of KRGSF on the expression of the genes encoding the proinflammatory mediators, *Cox2* and iNOS (Figure 6B). *Cox2* and iNOS mRNA expression was much higher in 12-month-old than in 6-month-old untreated mice, but this effect was ameliorated by the KRGSF treatment of 12-month-old mice. On the basis of these results, we hypothesize that aging is associated with increases in the expression of NLRP3 inflammasome components, which would cause ovarian dysfunction, but KRGSF ameliorates these changes.

## 4. Discussion

Previous studies have shown that ginseng extract has an anti-aging effect, improves ovulation, and prevents premature ovarian failure [31,32]. These results suggest that saponins, the main biologically active compounds in ginseng, may be effective at mitigating the effects of aging on ovarian health. In the present study, we have determined the effects of age and saponins on gene expression in mouse ovaries, and investigated how saponins might mitigate aging-related damage in the ovary. To determine the effect of aging on the ovaries of C57BL/6 mice, we compared gene expression in 12- and 6-month-old mice, and found that the expression of 236 genes was >1.5-fold higher, and that of 580 genes was >1.5-fold lower, in the ovaries of the older mice. The expression of 81 genes was significantly higher and that of 181 genes was significantly lower in the older mice. These data demonstrate an effect of aging on the gene expression profile of mouse ovaries.

We then investigated the effect of KRGSF treatment on the ovarian gene expression profile of 12-month-old mice, and found that the expression of 369 genes was >1.5-fold higher and that of 580 genes was >1.5-fold lower than in 6-month-old mice. Of these, the expression of 194 genes was significantly higher and that of 222 genes was significantly lower. Thus, KRGSF treatment also affects ovarian gene expression in mice. We next analyzed the expression data using GO enrichment analysis. The top 12 enriched molecular functions of the genes that were downregulated with age included oxidoreductase activity (GO:0016616), and this GO function was also included in the top 10 enriched molecular functions of the genes that were upregulated by KRGSF treatment.

*Idh1* was one of the genes included in the molecular function of oxidoreductase activity (GO:0016616) and its expression was affected by both aging and KRGSF treatment in the microarray analysis. Therefore, we performed quantitative RT-PCR to verify these findings, and indeed, compared with 6-month-old mouse ovaries, the mRNA expression of *Idh1* was significantly lower in 12-month-old mouse ovaries, but was increased by KRGSF treatment, such that there was no statistically significant difference between the expression in the KRGSF-treated 12-month-old mouse ovaries and the untreated 6-month-old mouse ovaries. These results suggest that KRGSF ameliorates the age-related reduction in the expression of genes encoding oxidoreductases, including that of *Idh1* [33].

Because *Idh1* is well known to inhibit intracellular oxidative stress, we next evaluated the potential effects of KRGSF on oxidative stress response by measuring the expression of key genes by qRT-PCR. Compared with 6-month-old untreated mice, the mRNA expression of antioxidant genes (*Nqo1*, *Gclm*, *Nrf2*, and *Nox2*) [34] and of gene encoding oxidant enzymes (*Sod2*, *Catalase*, *Gpx1*, and *Nlrp3*) [35] was much higher in the ovaries of 12-month-old untreated mice. However, KRGSF treatment reduced the expression of these genes, such that there were no significant differences from the expression levels in the 6-month-old untreated mice. These results suggest that KRGSF ameliorates the age-dependent accumulation of ROS, and may therefore prevent the development of oxidative stress response, which damages ovaries.

We also determined the effects of age and KRGSF treatment on the expression of genes encoding components of the NLRP3 inflammasome, which can be activated by ROS [12,29]. The genes encoding NLRP3, ASC, IL-1β, and caspase-1 were all expressed at higher levels in 12-month-old than in 6-month-old untreated mice, but these effects were ameliorated by KRGSF treatment. In addition, the expression of genes encoding the proinflammatory mediators COX-2 and iNOS were much higher in 12-month-old than in 6-month-old untreated mice, but this effect of aging was ameliorated by KRGSF treatment. On the basis of these results, we hypothesize that aging induces the expression of NLRP3 inflammasome components, which leads to ovarian dysfunction, but that KRGSF treatment may ameliorate these defects.

## 5. Conclusions

In the present study, we have identified evidence that KRGSF may inhibit the loss of ovarian function that results from aging-related oxidative stress response and NLRP3 inflammasome activation in mice. Our results have improved understanding of age-related ovarian damage, and we suggest that KRGSF has the potential to be used to alleviate the deterioration of female reproductive capacity due to aging.

## Figures and Tables

**Table 1:** List of PCR primers used in this paper.

Gene	Forward(5′-3′)	Reverse(5′-3′)
*Idh1*	CCAGTTTGAAGCTCAGAAGA	TACCGTCTTACCATCTGGAC
*Nrf2*	GCCCACATTCCCAAACAAGAT	CCAGAGAGCTATTGAGGGACTG
*Gclm*	GCATCACGATTCTCTATGCCTAA	TCCAGCTGTGCAACTCCAAGG
*Nqo1*	AGGATGGGAGGTACTCGAATC	AGGCGTCCTTCCTTATATGCTA
*Nox2*	GACCCAGATGCAGGAAAGGAA	TCATGGTGCACAGCAAAGTGAT
*Gpx1*	CTCTCCGCGGCGGCACAGT	CCGCCACCAGGTCGGACGTAC
*Gr*	CACGACCATGATTCCAGATG	CAGCATAGACGCCTTTGACA
*Sod2*	CTGAGGAGAGCAGCGGTCGT	CTTGGCCAGAGCCTCGTGGT
*Catalase*	TCCGAGATCTTTTCAATGCCATCG	TCGAGCGCGGTAGGGACAGTTCAC
*Nlrp3*	ACCAGCCAGAGTGGAATGAC	ATAGAGAGGGCGTAGAGGTA
*Asc*	TCACAGAAGTGGACGGAGTG	TCATCTTGTCTTGGCTGGTG
*pro-IL-1β*	TTGACGGACCCCAAAAGATG	AGGACAGCCCAGGTCAAAG
*IL-1β*	CAGGATGAGGACATGAGCAC	CTCTGCACACTCAAACTCCA
*pro-Caspase-1*	TGGTCTTGTGACTTGGAGGA	ACGACTCTTAGCACGGTTAT
*Caspase-1*	ATGCCGTGGAGAGAAACAAG	GGTGTTGAAGAGCAGAAAGCA
*iNOS*	AACGGAGAACGTTGGATTTG	CAGCACAAGGGGTTTTCTTC
*Cox2*	CACTACATCCAGACCCACTT	ATGCTCCTGCTTGAGTATGT
*18S*	CGGCGACGACCCATTCGAAC	GAATCGAACCCTGATTCCCCGTC
